# Muscle Protein Synthesis Responses Following Aerobic-Based Exercise or High-Intensity Interval Training with or Without Protein Ingestion: A Systematic Review

**DOI:** 10.1007/s40279-022-01707-x

**Published:** 2022-06-08

**Authors:** Reza Bagheri, Isabelle Robinson, Sajjad Moradi, Jessica Purcell, Elita Schwab, Tharindie Silva, Brooke Baker, Donny M. Camera

**Affiliations:** 1 Department of Exercise Physiology, University of Isfahan, Isfahan, Iran; 2 Department of Health Sciences and Biostatistics, Swinburne University, Room SPW224, Mail H21, PO Box 218, Hawthorn, VIC 3122 Australia; 3 Nutritional Sciences Department, School of Nutritional Sciences and Food Technology, Kermanshah University of Medical Sciences, Kermanshah, Iran

## Abstract

**Background:**

Systematic investigation of muscle protein synthesis (MPS) responses with or without protein ingestion has been largely limited to resistance training.

**Objective:**

This systematic review determined the capacity for aerobic-based exercise or high-intensity interval training (HIIT) to stimulate post-exercise rates of MPS and whether protein ingestion further significantly increases MPS compared with placebo.

**Methods:**

Three separate models analysed rates of either mixed, myofibrillar, sarcoplasmic, or mitochondrial protein synthesis (PS) following aerobic-based exercise or HIIT: Model 1 (*n* = 9 studies), no protein ingestion; Model 2 (*n* = 7 studies), peri-exercise protein ingestion with no placebo comparison; Model 3 (*n* = 14 studies), peri-exercise protein ingestion with placebo comparison.

**Results:**

Eight of nine studies and all seven studies in Models 1 and 2, respectively, demonstrated significant post-exercise increases in either mixed or a specific muscle protein pool. Model 3 observed significantly greater MPS responses with protein compared with placebo in either mixed or a specific muscle fraction in 7 of 14 studies. Seven studies showed no difference in MPS between protein and placebo, while three studies reported no significant increases in mitochondrial PS with protein compared with placebo.

**Conclusion:**

Most studies reporting significant increases in MPS were confined to mixed and myofibrillar PS that may facilitate power generating capacity of working skeletal muscle with aerobic-based exercise and HIIT. Only three of eight studies demonstrated significant increases in mitochondrial PS post-exercise, with no further benefits of protein ingestion. This lack of change may be explained by the acute analysis window in most studies and apparent latency in exercise-induced stimulation of mitochondrial PS.

## Key Points

**Table:** 

Systematic interrogation of MPS responses with or without protein ingestion has mostly been limited to resistance training.
From this systematic review, eight (of nine) and seven (of seven) studies demonstrated significant post-exercise increases in either mixed MPS or at least one pool of muscle proteins analysed in Models 1 (no protein ingestion) and 2 (peri-exercise protein ingestion but no placebo comparison), respectively. Compared with a non-protein placebo, only seven (of 14) studies in Model 3 showed protein ingestion induced significantly greater MPS responses in mixed MPS or at least one of the muscle protein pools measured.
The majority of studies collectively demonstrated aerobic-based exercise and HIIT, both with and without protein intake, significantly increased mixed and myofibrillar protein synthesis post-exercise which intuitively facilitate general muscle tissue remodelling processes. In contrast, and somewhat paradoxically, there was only limited evidence of increases in mitochondrial protein synthesis post-exercise or with protein ingestion.

## Introduction

Skeletal muscle proteins are constantly turned over to both synthesize new proteins for cellular function and remove damaged proteins caused by protein misfolding, oxidative stress, etc. [1]. Resistance training is well-established to stimulate both muscle protein synthesis (MPS) and muscle protein breakdown (MPB) that can result in a net positive protein balance provided there is ingestion of liquid- or solid-based protein sources during recovery from the exercise bout [2, 3]. In this regard, there is a synergistic increase in MPS when protein is ingested following resistance training compared with the MPS response of either protein or exercise independently [4–6]. The ensuing positive net protein balance provides the basis for muscle hypertrophy with repetitive bouts of resistance training and protein ingestion over the course of multiple weeks and months [7–9]. Specifically, exercise-induced and dietary protein-induced increases in MPS, rather than suppression of MPB, are considered to be the main determinant of changes in net protein balance crucial for enhancing muscle mass, function and quality [10].

At the opposite end of the exercise training continuum is aerobic-based exercise conventionally characterised by repetitive, low force muscle contractions/movements at submaximal exercise intensity (i.e., 45–75% maximal oxygen uptake [*V*O_2max_]) performed for sustained periods (i.e., 30 min or longer). Studies throughout the last half-century have consistently demonstrated aerobic exercise to enhance cardiovascular fitness [11] and improve oxidative capacity via a combination of cardiac (i.e., stroke volume), circulatory (i.e., oxygen-carrying capacity), and skeletal muscle adaptations [12]. Regarding skeletal muscle, previous works in animal and human studies have demonstrated increases in expression and abundance of mitochondrial enzymes/proteins, capillary density and mitochondrial capacity [12–14]. Similarly, high-intensity interval training (HIIT), defined as ‘near maximal’ intensity (85–95%) of maximal heart rate interspersed with short recovery blocks, has also been shown to promote cardiorespiratory, vascular and metabolic-related adaptations [11, 15]. Collectively, these adaptation responses are inversely associated with cardiovascular disease risk and all-cause mortality [16].

In the last decade, accumulating studies in both women and men demonstrate the potential for aerobic-based exercise and HIIT to promote anabolic adaptations, as evidenced by significant increases in leg lean mass, quadriceps muscle volume and size [17–23]. The basis for such anabolic responses intuitively centres on post-exercise increases in MPS. Indeed, an increasing number of studies have reported both aerobic-based exercise and HIIT to stimulate rates of MPS in younger and older adults [17, 18]. From a health perspective, such increases in MPS may not only assist in promoting and maintaining skeletal muscle mass and function with advancing age but also provide another anabolic stimulus in addition to resistance training, which remains the ‘gold standard’ for combatting sarcopenia despite its low participation and adherence rate in older adults [24, 25]. From a performance perspective, increases in MPS with aerobic exercise or HIIT may increase muscle power generating capacity and oxidative phosphorylation responses depending on whether myofibrillar or mitochondrial protein synthesis rates are stimulated, respectively. Similar to resistance training, protein ingestion may also stimulate MPS responses with aerobic-based exercise and HIIT, helping repair exercise-induced muscle damage [17, 26, 27]. Such responses may theoretically promote training adaptation responses while concomitantly enhancing skeletal muscle recovery and repair [26]. In support of this thesis, a recent meta-analysis of over 1100 participants from healthy and clinical populations showed protein supplementation to significantly enhance peak oxygen uptake and workload power compared with a placebo (protein-free) condition following chronic endurance training [28]. However, despite the multitude of studies in the last 2 decades investigating MPS responses following aerobic-based exercise or HIIT, no systematic consensus exists for the capacity for aerobic exercise or HIIT to increase rates of MPS based on available literature. The primary aims of this review were to systematically consolidate literature that has investigated rates of either mixed, myofibrillar, mitochondrial, or sarcoplasmic MPS responses: (1) following aerobic-based exercise or HIIT independent of protein or in studies including protein ingestion only (i.e., no comparison to a non-protein condition): and (2) to compare MPS responses in studies that incorporated protein and placebo conditions following aerobic-based exercise or HIIT.

## Methods

### Literature Search Strategy

This study was registered in PROSPERO (#CRD42021292287) and reported in accordance with the Preferred Reporting Items for Systematic Reviews and Meta-Analyses (PRISMA) guidelines [29]. The search strategy for this systematic review is based on the PRISMA 2009 guidelines, and a Population, Intervention, Comparison, Outcome, and Setting (PICOS) framework was used to determine the search strategy and study characteristics. A systematic literature search of the PubMed, Web of Science, MEDLINE, and Cochrane databases was performed, with the final literature search completed on 30 December 2021. These databases were selectively chosen due to their extensive coverage of original research articles in the areas of exercise, nutritional and clinical sciences. Search terms used were ‘muscle protein synthesis’, ‘protein synthesis’, ‘endurance’, ‘exercise’, ‘aerobic’, ‘high-intensity interval training’, ‘fractional synthesis rate’, ‘sprint interval training’, and ‘protein’. The Boolean operators ‘and’ and ‘or’ were used to combine search terms. Additional studies were identified through the reference lists of articles (e.g., reviews) from relevant fields of study. The systematic search for this study followed Table 1 and presents the PICOS parameters that were used to define the scope of the research included. No filters were applied to identify all relevant articles published on the topic.

**Table 1:** PICOS (Population, Intervention, Comparison, Outcome, Setting/Study Design) criteria for inclusion of studies

Parameter description
Population human studies in adults + 18 years of age
Intervention aerobic-based exercise or HIIT with or without protein ingestion
Comparison:
(1) pre- vs. post-exercise or post-training intervention
(2) post-exercise/training protein vs. placebo
Outcome rates of MPS (fractional synthetic rate)

Setting single and parallel group, and crossover studies

*HIIT* high-intensity interval training, *MPS* muscle protein synthesis

### Eligibility Criteria

#### Types of Studies and Participants

Single-group, parallel-group, and randomised or non-randomised crossover study (including placebo-controlled) designs that directly compared either (1) MPS responses between rest (i.e., pre-exercise) versus post-exercise (acute studies) or post-intervention (short-term or chronic studies), or (2) MPS responses between protein versus non-protein/placebo ingestion post-exercise (acute studies) or post-intervention (short-term or chronic studies) within the same study, all conducted in accordance with ethical standards, were eligible for inclusion. Studies were restricted to original manuscripts (not abstracts or reviews) written in English, and no publication date restrictions were applied. Studies that recruited healthy adults, both male and female, of all age ranges and aerobic fitness capacity were included in this systematic review. Studies where recruited participants had been diagnosed with compromised health conditions or chronic illnesses, including, but not limited to, cancer cachexia, cardiovascular or respiratory conditions, arthritis, and obesity or diabetes, or where participants were taking any medications that may produce hypo- or hyper-anabolic stimuli, were excluded. Furthermore, studies in which an aerobic exercise or HIIT bout composed part of a combined (‘concurrent’) exercise session were excluded due to subsequent effects on MPS responses not being able to be definitively isolated to only the aerobic or HIIT component. Young adults were defined in the age range of 18–34 years, middle-aged in the range of 35–59 years, and older adults in the age range of > 60 years.

#### Types of Interventions

Studies utilising a single bout (defined herein as acute), short-term (defined herein as 0.5–10 weeks exercise training duration), or chronic (defined herein as 10 + weeks exercise training duration) aerobic-based exercise or HIIT stimulus and/or protein ingestion (defined below) were included in this systematic review. Aerobic-based exercise stimuli herein are defined by any intervention that incorporated low-to-moderate intensity or medium-to-high duration endurance training (e.g., walking, cycling, running, leg kicks). HIIT stimuli herein are defined by any intervention that incorporated brief and repetitious periods (≤ 4 to 10 min) of intense continuous exercise (80–100% peak heart rate [HRpeak]) interspersed with short periods of rest or recovery (sprint interval training [SIT], repeated Wingate sprints, step-ups]. Studies that incorporated a protein condition/ingestion in this systematic review were those that included complete, isolate, hydrolysed or concentrate protein sources, isolated amino acid sources, or combined complete and isolated sources. Examples included whey, casein, calcium caseinate, whole milk, skim milk, almond milk, essential amino acid (EAA), leucine, lactalbumin and collagen peptides. Additionally, to help delineate and improve clarity regarding which studies are deemed to include a protein condition in their design, a cut-off of a 4-h peri-exercise window (i.e., 4 h prior to, during, or post-exercise) was applied based on available information provided within the manuscript. Studies in which protein/amino acids were co-ingested with carbohydrates and/or fats were also included. Placebo/control comparisons with protein administration included any protein-free conditions comprising carbohydrate, artificial sweetener, or water.

#### Types of Outcome Measures

The primary outcome measure from eligible studies was a qualitative assessment based on statistical outcomes for measurements of post-exercise rates of MPS in response to aerobic-based exercise with or without protein ingestion. All included studies were required to assess MPS via calculation of the muscle fractional synthetic rate (FSR) using the precursor product model. The precursor-product model measures the rate at which a tracer is incorporated into bound muscle protein between sequential muscle biopsies over a specified period of time and is considered the gold standard for assessing in vivo MPS in humans [30, 31]. Furthermore, this approach allows MPS assessment within specific protein subfractions (i.e., myofibrillar, mitochondrial, and sarcoplasmic). Therefore, any studies that used the two- or three-pool arteriovenous balance method (indirect estimate of MPS) were excluded, as well as those that investigated whole-body measures of protein synthesis.

### Data Collection and Analysis

#### Selection of Studies

Eligibility appraisal of the titles and abstracts generated by the literature search was conducted independently by three reviewers (IR, BB, DMC). All titles and abstracts deemed ineligible were excluded, while those determined to be potentially eligible for inclusion in the systematic review were reserved and the full-text articles were obtained for further screening. Full-text articles were subsequently screened by three independent reviewers (RB, JP, DMC) for relevance using the eligibility criteria described above. Any disagreements between the two reviewers were resolved by consensus. All records generated by the literature search of the PubMed, Web of Science, MEDLINE, and Cochrane databases were managed using the reference management software package EndNote (Thomson Reuters, v.X8). Duplicate records were removed using the ‘find duplicates’ function in Endnote.

#### Data Extraction and Management

Three reviewers (IR, BB, DMC) independently extracted all data (i.e., study characteristics, exercise and protein intervention parameters, changes in rates of MPS, and outcome data) from all included studies using a customised data extraction table. Any disagreements were resolved by consensus between the three reviewers. Categories of data extracted included (1) participant characteristics (e.g., age, number of participants, sex, relative maximal oxygen consumption (*V*O_2max_) if measured, and physical activity status; (2) exercise intervention (e.g., exercise mode, exercise intensity, exercise duration, exercise frequency/weekly training schedule if applicable); (3) protein intervention (protein type, dose, timing of ingestion, co-ingested macronutrients) if applicable; and (4) timing of muscle biopsies and MPS assessment (e.g., measurement period, mixed or muscle subfraction: myofibrillar, sarcoplasmic or mitochondrial) and data outcome details (i.e., qualitative appraisal of statistically significant changes in MPS response).

#### Quality Assessment

The Revised Cochrane risk of bias tool for randomised trials (ROB2) was used to assess the methodological quality of each included randomised controlled trial [32] by two reviewers (SM and RB). Additionally, the Risk Of Bias In Non-randomized Studies of Interventions (ROBINS-I) tool was applied by the same two reviewers to evaluate the methodological quality of each included non-randomised controlled trial [33].

#### Method of Data Synthesis

Consistent with previous and relevant systematic review methodology [31, 34], data from included studies were qualitatively synthesized due to the heterogeneous experimental methodology employed when assessing MPS (e.g., amino acid stable isotope tracer, deuterium oxide tracer, muscle protein subfraction analysis, duration of tracer incorporation, and precursor pool) between different studies, which can result in varying rates of MPS between studies [35]. Post data extraction, three separate models were constructed to compare the potential for aerobic-based exercise or HIIT with or without protein ingestion to alter MPS. Model 1 was composed of studies that incorporated aerobic-based exercise or HIIT as the only form of stimulus (i.e., no peri-exercise protein ingestion), and Model 2 included studies that measured rates of MPS following aerobic-based exercise or HIIT that also included peri-exercise protein ingestion with no placebo intervention/comparison. To appropriately determine the capacity for Models 1 or 2 to significantly alter the rates of MPS, eligible studies were required to include measures of MPS at rest (i.e., basal levels), as well as post-exercise (acute studies)/intervention (short-term or chronic studies) rates of MPS for direct comparison. While there is no direct protein versus placebo comparison in studies comprising Model 2, these studies were included to provide a more comprehensive appraisal of literature associated with measuring MPS responses in human skeletal muscle following aerobic-based exercise or HIIT. Model 3 included studies that incorporated placebo-controlled designs where post-exercise (acute studies)/intervention (short-term or chronic studies) rates of MPS were directly compared between protein ingestion and placebo/protein-free conditions following aerobic-based exercise or HIIT.

## Results

### Literature Search

**Fig. 1:**
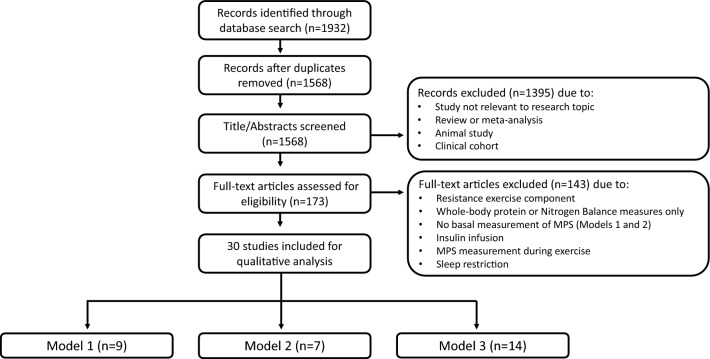
Screening process for identifying and selecting eligible studies. *MPS* muscle protein synthesis

Figure 1 displays the screening process for selecting eligible studies for inclusion into the systematic review. A total of 1932 records were produced by the literature search, of which 1568 records remained following the removal of duplicates. Following the screening of titles and abstracts, 1395 records were excluded, largely due to the presence of study records irrelevant to the research topic, as well as articles that were reviews or meta-analyses or conducted in animals or clinical cohorts (e.g., cancer cachexia). Overall, 173 articles were determined to be potentially relevant based on the information in the abstract and were then fully read for assessment of eligibility. After full-text reading, 143 articles were excluded, largely due to incorporating resistance training or concurrent resistance and aerobic/HIIT exercise. Further full-text articles were also excluded due to measures of whole-body protein synthesis only or use of nitrogen balance, no basal measurement of MPS for Models 1 and 2 [36–41], insulin infusion [42], measurement of MPS during aerobic-based exercise [43], and a study design in which participants performed an HIIT in a sleep-restricted state, which was deemed to be a non-comparable factor to other included studies [44]. Accordingly, a total of 30 studies met the eligibility criteria and were thus included for qualitative analysis (Fig. 1).

### Included Studies

Among the selected studies, there was a large degree of heterogeneity related to the age and fitness characteristics of participants; the type, intensity, and duration of aerobic exercise or HIIT interventions included in the study designs; and the types of protein ingested. Table 2 presents the summary of findings from studies that measured rates of MPS at rest and following acute, short-term, and chronic aerobic-based exercise or HIIT alone (i.e., no peri-exercise protein ingestion). Table 3 presents the summary of findings from studies that measured rates of MPS at rest and following acute, short-term, and chronic aerobic-based exercise or HIIT with protein-only conditions (i.e., no placebo comparison). Table 4 summarises the findings from studies that compared rates of MPS following acute, short-term and chronic aerobic exercise or HIIT between protein and placebo/protein-free interventions.

**Table 2:** Studies in Model 1 investigating the rates of muscle protein synthesis following aerobic-based exercise or HIIT as the only form of stimulus (i.e., no peri-exercise protein ingestion)

Study (PMID)	Sex and age; years	Training status *V*O_2max_ (mL/kg/min)	Exercise intervention (mode, duration, intensity)	Muscle biopsy timepoints	Muscle subfraction(s)	Main outcomes
*Acute*						
Miller et al. [65] (16,002,437)	M (*n* = 8); ~ 25 years	Recreational NA	One-legged kicking; 60 min; 67% *W*_max_	Rest, + 24 h, + 48 h, + 72 h	Myo and Sarco	Myo: ↑ 6 h, 24 h, 48 h, 72 h Sarco: 6 h: NA; ↑ 24 h, 48 h; ↔ 72 h
Miller et al. [45] (16,131,512)	F (*n* = 16); ~ 26 years	Recreational NA	One-legged kicking; 60 min; 67% *W*_max_	Rest, + 24 h	Myo	↑ 24 h
Harber et al. [73] (19,118,097)	M (*n* = 8); ~ 26 years	Trained ~ 63 mL/kg/min	Treadmill; 45 min; 75% *V*O_2max_	Rest, + 24 h	Mixed	*Soleus*: ↑ 24 h *Vastus Lateralis*: ↑ 24 h
Mascher et al. [68] (21,385,328)	M (*n* = 16); ~ 25 years	Recreational NA	One-legged kicking; 60 min; ~ 65–70% *V*O_2max_	Rest, 0 min, + 90 min OR + 180 min	Mixed	↔ 0–90 min ↑ 90–180 min
Di Donato et al. [67] (24,595,306)	M (*n* = 8); ~ 21 years	Recreational ~ 47 mL/kg/min	Cycling; 30 min @ 60% *W*_max_ OR 60 min @ 30% *W*_max_	Rest; + 30 min, + 4.5 h, + 24 h, + 28 h	Myo and Mito	Myo: ↑ 0.5–4.5 h; 24–28 h Mito: ↔ 0.5–4.5 h; ↑ 24–28 h
Bell et al. [47] (25,650,305)	M (*n* = 22); ~ 67 years	Sedentary NA	Cycling: 10 × 1 min @ 90% *V*O_2max_ OR 30 min aerobic cycling: 30 min @ 55–60% *V*O_2max_	Rest, + 24 h, + 48 h	Myo and Sarco	HIIT: Myo ↑ 24 h, 48 h; Sarco ↑ 24 h, ↔ 48 h Aerobic: Myo ↔ 24 h, 48 h; Sarco ↔ 24 h, 48 h
Serrano et al. [46] (34,408,662)	F (*n* = 3), M (*n* = 4); ~ 29 years	Recreational ~ 39 mL/kg/min	Cycling; 45 min; 65% HRR	Rest, + 3.15 h	Mixed and Mito	Mixed: 3.15 h ↔ Mito: 3.15 h ↔
*Chronic*						
Short et al. [48] (14,506,079)	F (*n* = 18), M (*n* = 17); 19–87 years	Recreational NA	Cycling; 20 min; 70% max. HR; 3 sessions/week; 20 weeks	72–96 h post final session	Mixed	↑ 72–96 h
Brightwell et al. [49] (31,123,725)	F (*n* = 8), M (*n* = 4); ~ 73 years	Sedentary ~ 23 mL/kg/min	Treadmill; 45 min; 70% HRR; 3 days/week; 24 weeks	24 post final session	Myo	↑ 24 h

*↑* indicates significant (*p* < 0.05) increase post-exercise, ↔ indicates no change, + indicates post-exercise, *F* females, *M* males, *Myo* myofibrillar, *Sarco* sarcoplasmic, *Mito* mitochondrial, *HIIT* high-intensity interval training, *HRR* heart rate reserve, *W*_*max*_ maximal workload, *NA* not available/applicable, *VO*_*2max*_ maximal oxygen uptake, *max.* maximum, *HR* heart rate

**Table 3:** Studies in Model 2 investigating rates of MPS following aerobic-based exercise or HIIT that also included a peri-exercise protein nutritional component with no placebo intervention/comparison

Study (PMID)	Sex and age; years	Training status *V*O_2max_ (mL/kg/min)	Exercise intervention (mode, duration, intensity)	Muscle biopsy timepoints	Muscle subfraction(s)	Protein source	Protein amount	Co-ingested nutrients	Main outcomes
*Acute*									
Cuthbertson et al. [50] (16,263,770)	M (*n* = 8); ~ 25 years	Recreational NA	Step-ups; 6 × 3 min; NA	Rest, 0 h, + 3 h, + 6 h, + 24 h	Myo and Sarco	EAA	45 g	135 g sucrose	Myo: ↔ 3 h, ↑ 6 h, 24 h; sarco: ↔ 3 h, ↑ 6 h, 24 h
Donges et al. [51] (22,492,939)	M (*n* = 8); ~ 53 years	Sedentary ~ 39 mL/kg/min	Cycle; 40 min; 55% *V*O_2peak_	Rest, + 1 h, + 4 h	Myo and Mito	WPI	20 g	NA	Myo: 1–4 h ↔ Mito: 1–4 h ↑
Timmerman et al. [70] (22,572,647)	F (*n* = 3), M (*n* = 3); ~ 70 years	Sedentary NA	Treadmill; 45 min; 60–70% HRR	Rest, + 1.5 h, + 3 h	Mixed	EAA	20 g	35 g CHO	↑ 1.5–3 h (*p* < 0.06)
Abou Sawan et al. [71] (29,512,299)	M (*n* = 8); ~ 25 years	Trained ~ 62 mL/kg/min	Treadmill; 60 min; 69% *V*O_2peak_	Rest, + 5 h	Myo and Mito	Egg	18 g	60 g CHO, 17 g fat	Myo: ↑ 0–5 h Mito: ↔ 0–5 h
*Short-term*									
Wilkinson et al. [69] (18,556,367)	M (*n* = 10); ~ 20 years	Recreational ~ 43 mL/kg/min	One-legged cycling; 45 min; 75% *V*O_2peak_; 3 times/week; 10 weeks	Rest, + 4 h	Myo and Mito	Whey	1.1 g/kg body mass	~ 4.6 g/kg body mass CHO	Myo: ↔ basal, + 4 h Mito: ↔ basal, ↑ + 4 h
Oikawa et al. [52] (33,323,831)	F (*n* = 6), M (*n* = 5) ~ 24 years	Trained ~ 69 mL/kg/min	HIIT cycle; 4 × 4 min; ~ 70% of PPO; 3 sessions/week; 2 weeks	Rest, ~ 24 post final session	Myo and Sarco	LA or CP	LA: 19.7 g EAA: 4.3 g Leu; CP: 6.4 g EAA: 1.1 g Leu (per 40 g)	NA	Myo: ↑ LA and CP; ↑ LA to CP; Sarco: ↑ LA and CP; ↑ LA to CP
Stokes et al. [72] (34,610,129)	F (*n* = 22); ~ 66 years (three groups)	Recreational NA	Walking; ~ 150% of habitual daily steps; NA; daily for 3 days	Rest; post 3-d pro; post 3-d pro + walking	Myo	Whole milk; skim milk; almond milk	Whole and skim milk: 16 g/day; almond: 2 g/day	NA	↑ Post 3-d pro + walking

↑ indicates significant (*p* < 0.05) increase post-exercise, ↔ indicates no change, + indicates post-exercise, *F* female, *M* male, *Myo* myofibrillar, *Sarco* sarcoplasmic, *Mito* mitochondrial, *HIIT* high-intensity interval training, *HRR* heart rate reserve, *NA* not available/applicable, *EAA* essential amino acids, *Leu* leucine, *CHO* carbohydrate, *WPI* whey protein isolate, *LA* lactalbumin, *CP* collagen peptides, *VO*_*2max*_ maximal oxygen uptake, *VO*_*2peak*_ peak oxygen, *PPO* peak power output, *MPS* muscle protein synthesis

**Table 4:** Studies in Model 3 investigating MPS following acute, short-term and chronic aerobic exercise or HIIT between exogenous protein and placebo/protein-free interventions

Study (PMID)	Sex and age; years	Training status *V*O_2max_ (mL/kg/min)	Exercise intervention (mode, duration, intensity)	Muscle biopsy timepoints	Muscle subfraction(s)	Protein source	Protein amount	Co-ingested nutrients	Placebo	Main outcomes (protein vs. placebo)
*Acute*										
Howarth et al. [75] (19,036,894)	M (*n* = 6); ~ 22 years	Recreational NA	HIIT cycle; 12 × 10 min; 50–80% *V*O_2peak_	0 h, + 4 h	Mixed	Whey	0.4 g/kg	1.2 g/kg CHO	Low CHO: 1.2 g/kg, OR High CHO: 1.6 g/kg	↑ 0–4 h vs. low CHO and high CHO
Harber et al. [66] (20,720,176)	M (*n* = 8); ~ 25 years	Recreational ~ 52 mL/kg/min	Cycle; 60 min; 70% *V*O_2max_	Rest, + 2 h, + 6 h	Mixed	Whey and casein	0.37 g/kg	0.83 g/kg/ CHO; 0.03 g/kg/fat	Non-caloric placebo	↔ 2–6 h vs. non-caloric placebo
Breen et al. [55] (21,746,787)	M (*n* = 10); ~ 29 years	Trained ~ 66 mL/kg/min	Cycle; 90 min; 77% *V*O_2max_	0 h, + 4 h	Myo and Mito	Whey	20.4 g	50.8 g CHO	50.4 g CHO	Myo: ↑ 0–4 h vs. 1.0 g/kg/h CHO Mito: ↔ 0–4 h vs. 1.0 g/kg/h CHO
Coffey et al. [56] (21,165,642)	M (*n* = 8); ~ 21 years	Recreational ~ 51 mL/kg/min	HIIT cycle; 10 × 6 s; maximal	Rest, + 15 min, + 4 h	Myo and Mito	Whey + Leu	24 g whey + 4.8 g Leu	50 g CHO	Artificial sweetener + colour	Myo: ↑ 15 min–4 h vs. 50 g CHO Mito: ↔ 15 min–4 h vs. 50 g CHO
Hulston et al. [54] (21,364,482)	M (*n* = 8); ~ 22 years	Trained NA	Cycle; 180 min; 60% *V*O_2max_	Rest, 0 h, + 3 h	Mixed	Whey	0.16 g/kg	0.49 g/kg CHO	0.49 g/kg CHO	↑ 0–3 h vs. 0.49 g/kg CHO
Lunn et al. [57] (21,904,247)	M (*n* = 8); ~ 23 years	Trained ~ 53 mL/kg/min	Treadmill; 45 min; 65% *VO*_2peak_	Rest, 0 h, + 3 h	Mixed	Whey and casein	16 g	CHO	74.g CHO	↑ 0–3 h vs. 74 g CHO
Andersen et al. [74] (25,411,362)	F (*n* = 3), M (*n* = 5); ~ 33 years	Untrained NA	One-legged kicks; 40 min; 70% *V*O_2Max_	Rest, + 3 h	Mixed	Whey and casein	32.8 g protein	36.6 g CHO, 1.4 g fat	Fasted	↔ 0–3 h vs. fasted
Pasiakos et al. [39] (26,474,292)	F (*n* = 3), M (*n* = 37); ~ 22 years	Recreational ~ 50 mL/kg/min	Treadmill; 90 min; NA	Rest, + 15 min, + 195 min	Mixed; Myo; Sarco	EAA	10 g EAA (3.6 g Leu)	46 g CHO	5 g CHO	Mixed; Myo; Sarco: ↔ 15 min–195 min vs. 5 g CHO
Rowlands et al. [59] (25,026,454)	M (*n* = 12); ~ 30 years	Trained ~ 60 mL/kg/min	HIIT cycling; 2–8 × 2 min; 70–100% *W*_max_	Rest; + 30 min; + 240 min	Myo	EAA	170 g protein (15 g Leu) or 23 g protein (5 g Leu)	180 g CHO, 30 g fat	274 g CHO, 30 g fat	↑ 30–240 min with both protein beverages vs. 274 g CHO, 30 g fat
Rundqvist et al. [60] (28,860,165)	F (*n* = 3), M (*n* = 9); ~ 26 years	Recreational NA	Cycling (HIIT); 3 × 30 s; maximal	80 min; + 200 min	Mixed	EAA	300 mg/kg bw	1 g maltodextrin/kg	Artificial sweetener	↔ 80–200 min vs. artificial sweetener (muscle Phe enrichment); ↑ 80–200 min vs. artificial sweetener (plasma Phe enrichment)
Churchward-Venne et al. [61] (32,359,142)	M (*n* = 48); ~ 27 years	Trained ~ 58 mL/kg/min	Cycling; 90 min; 60% *W*_max_	0 h, + 3 h, + 6 h	Myo and Mito	Whey and casein	15 g OR 30 g OR 45 g	45 g CHO	45 g CHO	Myo: ↑ 0–6 h vs. 45 g CHO; Mito: ↔ 0–6 h vs. 45 g CHO
Larsen et al. [62] (31,992,300)	M (*n* = 9); ~ 28 years	Trained ~ 60 mL/kg/min	Cycling; 90 min; 65% *V*O_2peak_	Pre, 0 h, + 1 h, + 4 h	Myo	WPH	0.5 g/kg bw	0.06 g/kg bw CHO	Flavoured water	↔ 0–4 h vs. flavoured water
*Short-term*										
Robinson et al. [63] (21,613,572)	F (*n* = 13), M (*n* = 7); ~ 50 years	Sedentary ~ 25 mL/kg/min	Treadmill; 30 min; 65–85% *V*O_2 Max_; 3/week; 6 weeks	Pre; + 6 weeks	Mixed	Whey and casein	20 g	CHO	300 kcal CHO	↔ Post-intervention vs. 300 kcal CHO
*Chronic*										
Markofski et al. [64] (29,750,251)	F (*n* = 30), M (*n* = 15); ~ 72 years	Sedentary NA	Walking; 40 min; NA; 24 weeks	1 h; + 3 h	Mixed	EAA	15 g	NA	Artificial sweetener	↔ Post-intervention vs. artificial sweetener (basal rates of MPS); ↑ 60–180 min pre- and post-intervention in EAA + AE only

↑ indicates significant (*p* < 0.05) increase post-exercise, ↔ indicates no change, + indicates post-exercise, *F* female, *M* male, *Myo* myofibrillar, *Sarco* sarcoplasmic, *Mito* mitochondrial, *AE* aerobic exercise, *HIIT* high-intensity interval training, *W*_*max*_ maximal work load, *NA* not available/applicable, *bw* body weight, *EAA* essential amino acids, *Leu* leucine, *CHO* carbohydrate, *WPH* whey protein hydrolyte, *Phe* phenylalaine, *MPS* muscle protein synthesis, *VO*_*2max*_ maximal oxygen uptake, *VO*_*2peak*_ peak oxygen

### Quality of Studies

Table 5 illustrates the risk of bias assessment of the included studies. Our scoring showed that 20 studies were of high quality [45–64] with a low risk of bias. In addition, eight studies [65–72] were of medium quality with a moderate risk of bias, whereas two studies [73, 74] were of low quality with a serious risk of bias.

**Table 5:**
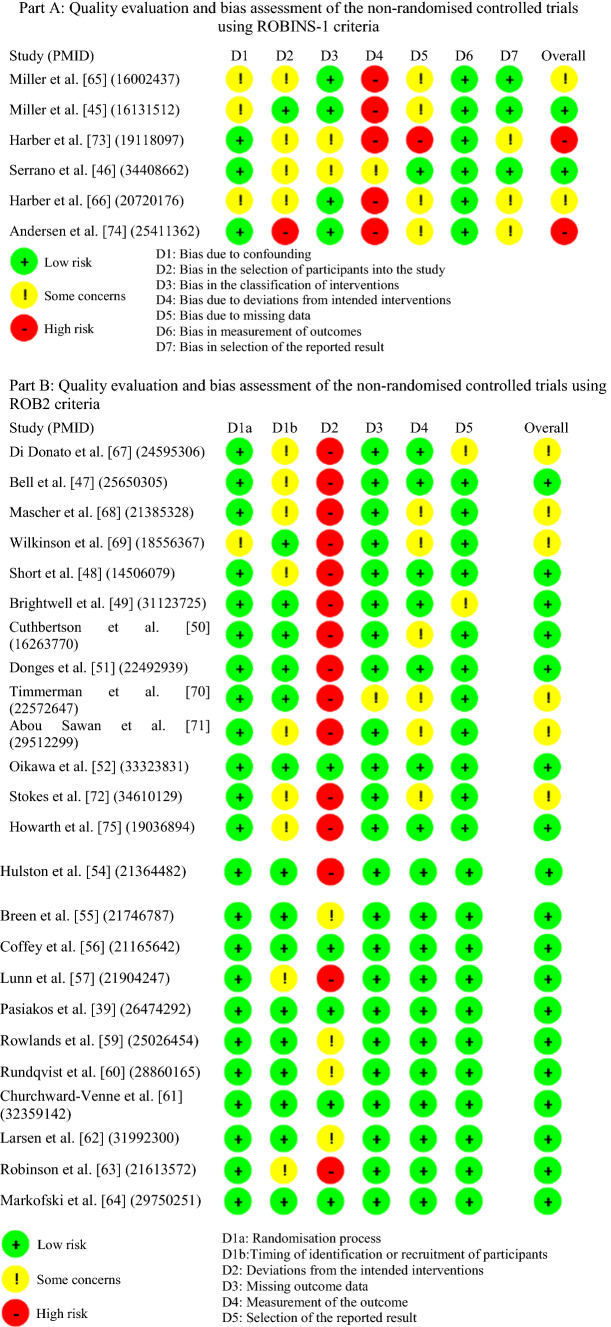
Quality evaluation and bias assessment of the included studies

### Participants

Despite different physical activity/exercise training histories, participants across all studies were healthy, with no reported metabolic conditions, smoking, or excessive alcohol use. Of the included studies, 18 recruited males only [47, 50, 51, 54–57, 59, 61, 62, 65–69, 71, 73, 75], 2 included females only [45, 72], and 10 included both males and females [46, 48, 49, 52, 58, 60, 63, 64, 70, 74]. Overall, 319 males and 128 females were recruited in eligible studies (Model 1: 87 males and 45 females; Model 2: 42 males and 31 females; Model 3: 190 males and 52 females). The age range of recruited participants from eligible studies was between 20 and 73 years. There were 22 interventions with young adults (mean age 25 years) compared with three and five interventions in middle-aged (mean age 52 years) and older adults (mean age 70 years). Regarding fitness level/physical activity status, eight studies were conducted in highly trained individuals (mean relative *V*O_2max_ of seven studies: 63.1 mL/kg/min); 15 were conducted in ‘recreationally’ trained participants (mean relative *V*O_2max_ of seven studies: 49.1 mL/kg/min), and seven were conducted in sedentary or untrained but otherwise healthy individuals (mean relative *V*O_2max_ of two studies: 31.0 mL/kg/min).

### Exercise Stimuli

Of the 30 studies, 23 incorporated an acute exercise study design, four incorporated a short-term study design, and three incorporated a chronic study design. Of the 23 acute study designs, cycling was utilised in 13 studies (Model 1: three; Model 2: one; Model 3: nine), treadmill in five studies (Model 1: one; Model 2: two; Model 3: two), one-legged kicking in four studies (Model 1: three; Model 3: one) and step-ups in one study (Model 2: one). Of the four short-term study designs, cycling was utilised in two studies (Model 2: two), treadmill in one study (Model 3: one) and walking in one study (Model 2: one). Of the three chronic study designs, treadmill was incorporated in one study (Model 1: one), cycling in one study (Model 1: one), and walking was incorporated in the other study (Model 3: one). Of the 30 eligible studies, 25 were aerobic-based stimuli (Model 1: eight; Model 2: six; Model 3: 11) with ranges in duration and intensity of 20–180 min (average: 58 min; mode: 45 min and 60 min) and 55–75% *V*O_2max_ (average: 69% *V*O_2max_; mode: 70% *V*O_2max_). Four studies were HIIT-based with ranges in the number of work bouts and their duration of 3–12 (average: 7) and 6 s–10 min (average: 3 min, 26 s), respectively. One study included both aerobic- and HIIT-based exercises in their study design [47].

### Nutritional Protein Intervention

Of the 21 studies (Model 2: 7; Model 3: 14) that incorporated a form of protein ingestion, six were EAA/leucine rich-based (absolute range: 10–45 g), seven were milk/whey-casein based (absolute range: 15–45 g), and six were whey-based-only beverages (absolute amount: 10–24 g). Egg and lactalbumin or collagen peptide sources were also used (*n* = 1 each). Studies reported relative protein ingestion ranging from 0.16 to 1.1 g/kg of protein.

### Experimental Methodology

Experimental methodology between studies was highly variable regarding the time period over which MPS was measured as well as the different muscle protein pools analysed. Of the 30 studies, five were single-group [48, 50, 65, 69, 73], 10 were parallel-group [45–47, 49, 58, 61, 63, 64, 68, 72], and 15 implemented a crossover design [51–57, 59, 60, 62, 66, 67, 70, 71, 74], of which 10 were placebo-controlled. Regarding the measurement of rates of MPS, 12 studies measured MPS solely at the mixed protein level, seven studies concomitantly measured myofibrillar and mitochondrial fractions, five studies measured rates of MPS only in the myofibrillar fraction, and three studies concomitantly measured myofibrillar and sarcoplasmic fractions. Of the remaining three studies, rates of a combination of the following protein pools were measured: mixed and sarcoplasmic [47], mixed and mitochondrial [46], and mixed, mitochondrial, and cytoplasmic [58].

Muscle biopsies for measurement of MPS were obtained from the *vastus lateralis* in all studies, while one study concomitantly measured MPS in the *vastus lateralis* and *soleus* muscles [73]. Biopsy time points utilised for measuring rates of MPS ranged from immediately post-exercise to 72 h post-exercise. Most biopsies across the three models were obtained between 0 and 6 h post-exercise (*n* = 42 biopsies), with fewer biopsies between 6.5 and 24 h (*n* = 8 biopsies) and 24.5 h plus (*n* = 5 biopsies).

### Data Synthesis

Of the seven acute studies in Model 1, increases in MPS with aerobic-based (six studies) or HIIT (one study) were reported (Table 2). These increases in MPS were apparent from between 1 and 48 h post-exercise within mixed (three studies [47, 68, 73]), myofibrillar (three studies [45, 65, 67]) and sarcoplasmic (one study [47]) fractions. One study observed a selective increase in mixed muscle FSR compared with rest when measured between 90 and 180 min, but not 0 (i.e., immediately post-exercise) to 90 min following a bout of aerobic exercise [68]. Furthermore, another study reported selective increases in myofibrillar but not mitochondrial protein synthesis between 0.5 and 4.5 h [67]. The two chronic studies in Model 1 reported significant increases in myofibrillar [49] and mixed [48] protein synthesis, respectively, following 20–24 weeks of treadmill or cycle exercise undertaken three times per week (Table 2).

Of the four acute studies in Model 2, selective increases in rates of myofibrillar, but not sarcoplasmic or mitochondrial protein synthesis, was demonstrated in the 0–5 h post-exercise recovery period in two studies with EAA and egg protein ingestion, respectively (Table 3) [50, 71]. This contrasts with another acute study in Model 2 that reported exclusive increases in mitochondrial but not myofibrillar protein between 1 and 4 h following aerobic-based exercise and ingestion of 20 g of whey protein [51]. Nonetheless, increases in both myofibrillar and mitochondrial protein synthesis were reported 24–28 h following aerobic-based exercise and an overnight protein-containing meal [67]. Of the three short-term studies within Model 2, increases in the rates of myofibrillar protein synthesis were observed with HIIT and lactalbumin or collagen peptide supplementation [52], as well as with walking and milk (collapsed data of full-fat, skim and almond milk) supplementation [72]. Increased rates of mitochondrial but not myofibrillar protein synthesis were also apparent 4 h following an acute bout of aerobic-based exercise that proceeded 10 weeks of aerobic-based exercise (Table 3) [69]

Of the 12 acute study designs eligible in Model 3, selective results from seven studies (aerobic-based exercise: five studies; HIIT: two studies) demonstrated significantly greater increases in MPS with protein versus placebo/non-protein conditions (Table 4). Specifically, this included significantly greater increases in the rates of myofibrillar (four studies [55, 56, 59, 61]) and mixed (three studies [53, 54, 57]) protein synthesis with protein compared with placebo during the 0–6 h recovery period. Among the four studies reporting significant increases in the rates of myofibrillar protein synthesis during this recovery period were three studies that simultaneously observed unchanged rates of mitochondrial protein synthesis [55, 56, 61]. In contrast, five studies reported no significant difference with protein ingestion from sources including EAA, whey, and casein compared with placebo in rates of mixed [58, 60, 66, 74], myofibrillar [58, 62], or sarcoplasmic [58] protein synthesis (Table 4). One of these five studies observed no differences in post-exercise rates of MPS between EAA and placebo conditions with skeletal muscle tissue phenylalanine enrichment, however a significant difference in MPS with EAA above placebo was observed when plasma-bound phenylalanine enrichment was used for FSR calculations (Table 4) [60]. In the one short-term study comprising Model 3, similar rates of mixed MPS were reported between either a mixed milk protein or carbohydrate beverage consumed post-exercise following 6 weeks of aerobic-based exercise performed three times per week (Table 4) [63]. In the one chronic study comprising Model 3, increased rates of mixed MPS were reported following an acute exercise bout performed pre and post 24 weeks of walking-based exercise with the EAA condition only [64]. However, these acute increases in MPS with EAA and walking exercise were not statistically greater compared with the increases in the rates of MPS observed following the placebo and walking condition. Similarly. basal rates of mixed MPS post-walking intervention were not different between EAA and placebo conditions (Table 4) [64].

## Discussion

The aim of this systematic review was to determine the capacity for aerobic-based exercise or HIIT to stimulate rates of MPS and whether protein ingestion can further significantly enhance MPS responses compared with a placebo/non-protein condition. Currently, systematic interrogation of MPS responses with or without protein ingestion has been largely limited to resistance training, which is perhaps unsurprising given that both aerobic-based exercise and HIIT are not conventionally associated with muscle hypertrophy. Findings from Models 1 and 3 from this systematic review provide robust evidence for aerobic-based exercise and HIIT to stimulate post-exercise rates of MPS. In Models 2 and 3, protein ingestion was also shown to significantly increase MPS responses with both exercise modes, although the extent to which these increases were mediated by protein intake cannot be completely discerned in Model 2 without a placebo comparison. Nonetheless, these increases in MPS were equivocal, with multiple studies comparing MPS responses between protein and placebo conditions showing no further increase in the rates of MPS with protein ingestion. Additionally, the majority of studies reporting significant increases in MPS in this systematic review were confined to rates of mixed and myofibrillar protein synthesis, with only three of eight studies demonstrating significant post-exercise increases in mitochondrial protein synthesis.

### Model 1

Eight of the nine studies comprising Model 1 demonstrated increases in synthesis rates of at least one pool of muscle proteins analysed. Acutely, rates of myofibrillar protein synthesis were increased in all studies within this model. Myofibrillar proteins function as contractile elements [76], with their remodelling and chronic accrual following resistance training forming the basis for promoting muscle hypertrophy through their insertion within functioning sarcomeres [77]. Increases in muscle cross-sectional area and mass are more typically associated with resistance training compared with aerobic-based or HIIT, given the load-bearing nature of resistance training-induced contraction [13]. However, numerous studies have also reported that increases in myofibre and whole muscle size followed aerobic-based exercise training [17, 18]. Similarly, increases in lean/fat-free mass have been observed following HIIT [17]. It must be noted that many of these studies that have reported increases in muscle hypertrophy and lean/fat-free mass have been conducted in either sedentary or untrained older adults [19, 78–81]. Intuitively, these participant demographics are likely to have a greater ‘ceiling’ for observing increases in muscle hypertrophy in response to exercise-induced contraction compared with recreational or highly trained individuals who would commence an exercise intervention with higher baseline levels of lean/fat-free mass due to their pre-existing conditioning.

**Fig. 2:**
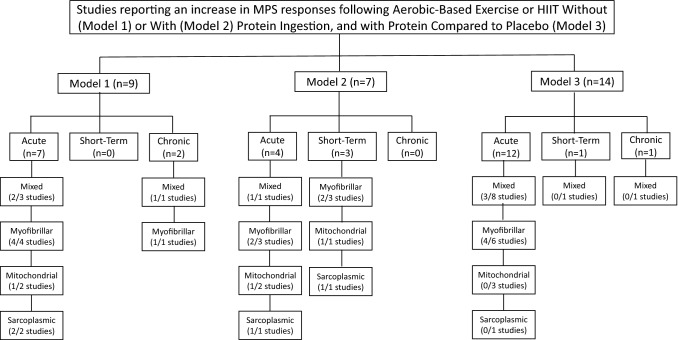
Summary of number of acute, short-term and chronic studies reporting a significant increase in either mixed, myofibrillar, mitochondrial or sarcoplasmic protein synthesis without (Model 1) or with (Model 2) protein ingestion, as well as with protein compared with a placebo condition (Model 3). *MPS* muscle protein synthesis, *HIIT* high-intensity interval training

The observed increases in the rates of myofibrillar protein synthesis within Model 1 were apparent, both acutely (i.e., post single bout) and following a 24-week intervention in response to different forms of training stress, including one-legged kicking, cycling and treadmill running. In addition to facilitating increases in muscle cross-sectional area, it has been put forward that increases in myofibrillar protein synthesis with aerobic-based exercise may also be important for facilitating the power generating capacity of working skeletal muscle [55]. Such potential power-based adaptation may also benefit sprinting, long jump, and javelin activities by helping promote an optimal power-to-body mass ratio to benefit performance [82]. While most studies in Model 1 demonstrating increases in myofibrillar protein synthesis included exercise protocols where the training intensity was at least 60% of work maximum (W_max_) (Table 2), the study by Di Donato and colleagues also reported significantly higher post-exercise rates of myofibrillar protein synthesis following cycling undertaken at only 30% of *W*_max_ [67]. Notably, this study observed acute (fasted) increases in myofibrillar protein synthesis following a single bout of cycling undertaken at either 30% or 60% of *W*_max_ during the acute (i.e., 0.5–4.5 h) post-exercise recovery window. In contrast, post-exercise (fed) myofibrillar synthesis rates were still significantly elevated 24–28 h following the cycling bout performed at 60% *W*_max_, but not 30% [67]. Four other studies in Model 1 that measured rates of myofibrillar or mixed protein synthesis following either a single bout of one-legged kicking at 67% *W*_max_ [45, 65], treadmill running at 75% *V*O_2max_ [73], or cycling at 60–90% *V*O_2max_ [47] also demonstrated significant increases 24 h or longer post-exercise. It could be speculated that such prolonged increases in MPS with aerobic-based exercise may be mediated by the potential for greater muscle damage induced in response to higher (compared with lower) workload intensities (Fig. 2).

### Model 2

Nutritional strategies to maximise recovery and subsequent exercise-induced adaptation response are widely used by recreational individuals along with elite athletes. The importance of EAA ingestion to maximise MPS responses with resistance training is well established [7, 83] and more recently following concurrent resistance and aerobic-based exercise [84]. In contrast, much less focus has been directed towards the effects of protein ingestion on MPS responses following aerobic-based exercise or HIIT, which, in some respects, is unsurprising considering the conventional paradigm that these exercise modes are not typically associated with skeletal muscle anabolism. Nonetheless, accumulating evidence is also starting to inform the importance of high-quality dietary protein to repair endurance training-induced muscle damage and promote MPS [18, 27]. Peri-exercise ingestion of EAA has been consistently shown to maximise MPS with resistance training [10, 85], although much less is known regarding their effects following aerobic-based exercise or HIIT. It has previously been shown that 10 g of EAA enriched with 3.5 g leucine ingested throughout moderate steady-state cycle exercise (60 min at 60% peak oxygen [*V*O_2peak_]) induced significantly higher rates of mixed MPS compared with an isonitrogenous EAA beverage with 1.87 g leucine [39]. Interestingly, this finding of enhanced stimulation of MPS with EAA that incorporates increased leucine content contrasts with findings involving resistance training [86, 87]. It is possible that the higher leucine availability with the leucine-enriched EAA ingested throughout the 1-h exercise bout in this study spared endogenous protein stores to a greater extent compared with the beverage with the lower leucine content. In support, others have also reported milk protein hydrolysate ingestion during endurance training to spare endogenous protein [88, 89]. Studies in Model 2 that provided participants with EAA contained totals of 45 g [50] and 20 g [70] EAA, which indicates that leucine ingestion of approximately 2.5 g (or higher) during or post moderate-intensity aerobic-based exercise can limit the dependence on endogenous protein and subsequently enhance MPS in recovery. While further studies are required to confirm this concept, additional support for higher leucine availability to augment MPS responses with short periods of intensified aerobic training was recently demonstrated in the study by Oikawa and colleagues comparing supplementation with α-lactalbumin or an isonitrogenous quantity of collagen peptides [52]. Rates of myofibrillar protein synthesis were significantly greater with daily supplementation of α-lactalbumin 60 g (comprising a total of 30 g EAA and 6.5 g leucine) compared with collagen peptides (9.6 g EAA and 2.2 g leucine) during intensified aerobic exercise (high-intensity training [HIT]: 4 × 4 min; three sessions) in endurance-trained participants (Table 3) [52]. Collectively, these findings suggest that, even in response to aerobic-based exercise or HIIT, protein quality may be an important consideration for the enhancement of skeletal muscle MPS and subsequently augmenting adaption responses to exercise training.

Another notable finding in the study by Oikawa and colleagues was that lactalbumin supplementation also significantly increased rates of sarcoplasmic protein synthesis, both above the resting/washout period as well as compared with collagen peptide supplementation [52]. Exercise-induced increases in sarcoplasmic protein synthesis represent remodelling of the non-myofibrillar pool of skeletal muscle protein; thus, alterations in this fraction are indicative of changes to cellular proteins implicated in mitochondrial biogenesis, substrate metabolism, and angiogenesis [69, 90]. Other studies within Model 2 also observed significant increases in the rates of either sarcoplasmic [50] or mitochondrial [51] protein synthesis.

Wilkinson and colleagues similarly reported increases in the rates of mitochondrial, but not myofibrillar, protein synthesis following 10 weeks of aerobic-based one-legged cycling (30–45 min at 75% *V*O_2peak_) [69]. In line with the classical exercise training principle of specificity [72], this response would indicate a ‘fine tuning’ of the skeletal MPS machinery to prioritise the synthesis of key metabolic enzymes and proteins required for the performance of the aerobic-based contractile activity. In this regard, it cannot be ruled out that the significant increases in rates of myofibrillar protein synthesis observed following acute/single bouts of aerobic-based exercise in all three models of this systematic review may be indicative of a non-specific or possible ‘stress’ response of working skeletal muscle to unfamiliar contractile-induced activity. Indeed, previous work has indicated a non-specificity in messenger ribonucleic acid (mRNA) and cell signalling responses to unaccustomed contractile stimuli [63, 73–75]. Nonetheless, it should also be pointed out that in the study by Wilkinson and colleagues, even though not statistically significant, the rates of myofibrillar protein synthesis were higher post-intervention with aerobic-based exercise. This would imply that myofibrillar proteins still play a role in mediating exercise adaptation responses with aerobic-based exercise.

In contrast, rates of mitochondrial protein synthesis were recently shown in another study within Model 2 to be unchanged when a mixed macronutrient meal consisting of 18 g of egg protein was ingested following a 60 min bout of treadmill running at 70% *V*O_2peak_ [71]. The authors postulated that the amount (and type) of protein provided to participants may have been inadequate to stimulate postprandial mitochondrial protein synthesis rates, particularly with the relatively high basal turnover rates in their recruited endurance-trained cohort [71]. In support of this premise, the same group previously showed ingestion of 18 g of egg protein was insufficient to replace oxidative losses associated with continuous, steady-state aerobic exercise (60 min treadmill running at 70% *V*O_2peak_) [91]. Multiple previous studies have demonstrated increased amino acid oxidation rates in response to aerobic-based exercise through stimulation of MPB rates [75, 92, 93]. Prolonged endurance training has been shown to preferentially oxidize branched-chain amino acids (BCAAs) [94, 95], while replacement of amino acid losses due to hepatic gluconeogenesis [96] provides further support for ingestion of high-quality, leucine-enriched protein during endurance training [27, 96]. Indeed, recent work in endurance athletes using the indicator amino acid oxidation method proposed an estimated average requirement and a recommended protein ingestion of 1.6 and 1.8 g.kg^−1^.d^−1^, respectively [97]. These collective findings suggest that the stimulation of sarcoplasmic protein synthesis following either endurance or HIIT may be dependent on the ingestion of sufficient quantities of high-quality protein so that exercise-induced oxidative losses do not depress rates of MPS. Nonetheless, it needs to be acknowledged that a severe limitation of studies included in Model 2 is that no direct comparison in MPS responses between protein and placebo/protein-free conditions are made. As such, the degree to which protein supplementation facilitated post-exercise/training increases in MPS in addition to the exercise stimulus can only be speculated.

### Model 3

Studies in Model 3 compared changes in MPS following aerobic-based exercise or HIIT between protein and placebo-controlled conditions. Findings from 8 of the 14 studies in this model demonstrated protein ingestion to induce significantly greater MPS responses compared with placebo/non-protein conditions in at least one of the muscle protein pools measured [53–57, 59, 61]. In contrast, seven studies showed no difference in MPS responses between protein and placebo [58, 60, 62–64, 66, 74], while another three studies showed no differences in rates of mitochondrial protein synthesis [55, 56, 61]. Interestingly, one of these seven studies reported a > 50% increase in MPS with whey and casein compared with placebo that was statistically non-significant, likely due to the *n* = 3 sample size [74]. Indeed, in this same study, the authors showed this beverage to induce a significant increase in MPS compared with a fasted placebo condition in a cohort of nine adults with muscle dystrophy [74]. Regardless, the equivocal nature of these findings with protein compared with placebo conditions is somewhat surprising when considering the multitude of studies that have reported significantly greater increases in MPS with protein ingestion compared with placebo following resistance training [10, 85]. One factor that may partly explain these results is the effects of nutrient co-ingestion. Most studies in Model 3 incorporated nutrient interventions whereby protein was co-ingested with carbohydrate, with absolute and relative quantities ranging between 45 and 50 g and 0.49 and 1.2 g/kg, respectively. The ingestion of such high amounts of carbohydrate following aerobic-based exercise or HIIT is unsurprising considering the established capacity for carbohydrates to direct glycogen resynthesis [98]. The replenishment of muscle glycogen stores is much less of a priority with resistance training considering the intermittent nature of this exercise modality, which likely explains why most exercise-nutrient studies involving resistance training do not co-ingest carbohydrates with protein. It has been shown that the bioavailability and digestibility of amino acids are slowed when co-ingested as part of a mixed meal that includes factors such as carbohydrate, fat, and dietary fibre [99, 100]. Thus, it is plausible that the co-ingested carbohydrate significantly delayed the digestion and subsequent absorption of amino acids to be made available for MPS. As a result, the 1–6 h post-exercise analysis window incorporated among the six acute studies that did not observe any differences between protein and placebo conditions may have been too short to observe potentially delayed increases in MPS with these nutrient protocols. Nonetheless, this does not explain why seven of the other acute studies showed statistically significant differences between protein and placebo conditions within this acute analysis window (Table 4). It is also unlikely that the quality and quantity of protein provided were factors in the studies that did not report significant differences in MPS between protein and placebo beverages. For instance, many of these studies provided high-quality biological protein sources such as whey and EAA and in amounts previously shown to maximise MPS, at least with resistance training. Further research that directly compares the effects of protein ingested independently or co-ingested with carbohydrates on rates of MPS following aerobic-based exercise or HIIT would provide new knowledge as to how carbohydrate co-ingestion may alter MPS.

Another notable finding from the studies in Model 3 was that none of the three studies that measured rates of mitochondrial protein synthesis showed any further enhancement in FSR with protein compared with placebo conditions. Intuitively, considering the established capacity for both aerobic-based exercise and HIIT to promote mitochondrial biogenesis and the stimulatory effects of protein on MPS, this finding was somewhat unexpected. Indeed, as identified in Models 1 and 2 in this systematic review, three of the six studies that measured the rates of mitochondrial protein synthesis did not observe any significant post-exercise increases in these protein fractions. One explanation for this is the timing of biopsies and post-exercise window for analysing MPS. As previously mentioned, most of the studies in this systematic review that measured rates of mitochondrial (or sarcoplasmic) protein synthesis were confined to 1–6 h. This time frame may be too premature for observing significant alterations in mitochondrial protein synthesis. Support for this premise comes from a study in Model 1, which showed rates of mitochondrial protein synthesis were unchanged 0.5–4.5 h following a bout of cycle exercise but were significantly elevated when analysed 24–28 h post-exercise [67]. From a molecular/mechanistic basis, previous work has shown that mRNA expression of peroxisome proliferator-activated receptor-gamma coactivator-1 alpha (PGC-1α), long considered the ‘master regulator’ of mitochondrial biogenesis, was elevated 6 h following a 1-h bout of cycling performed at 70% of *V*O_2max_ that proceeded a 2-week dietary intervention in which participants ingested 0.7 g/kg of a whey protein isolate supplement [101]. In contrast, previous work has demonstrated increased PGC-1α mRNA abundance 3–4 h post aerobic exercise or HIIT [102], which falls well within the 6 h timeframe studies in this systematic review used to measure mitochondrial protein synthesis. Additionally, coupling changes in mitochondrial protein synthesis with PGC-1α mRNA should be undertaken with caution [103], particularly considering PGC-1α can mediate other cellular responses following exercise [104]. Based on most findings from this systematic review, there is an apparent latency in stimulating mitochondrial protein synthesis compared with exercise-induced alterations in myofibrillar (and mixed) MPS. Thus, the delayed digestion and absorption of protein with carbohydrate co-ingestion that appears apparent in many of the studies in this systematic review may actually be beneficial for enhancing the delayed response in mitochondrial/sarcoplasmic protein synthesis by prolonging the length of time of elevated amino acid availability. Moreover, it has been purported that slower-release proteins (i.e., casein) may be a preferred choice for enhancing post-exercise rates of mitochondrial protein synthesis [26]. Either way, considering that the enhanced sensitivity of MPS to protein ingestion following resistance training has been shown to persist for 24–48 h post-exercise [105], it is possible the stimulatory effects of protein ingestion on mitochondrial protein biogenesis may similarly be apparent at these later time points.

## Conclusions and Future Directions

This is the first systematic review to focus on MPS responses following non-resistance training modalities, principally HIIT and aerobic-based exercise. Findings from this systematic review demonstrate the capacity for both HIIT and aerobic-based exercise to significantly enhance the rates of MPS, particularly mixed and myofibrillar proteins. The collective evidence from the three different models also showed equivocal results regarding post-exercise increases in mitochondrial (and to a lesser extent, sarcoplasmic) protein synthesis, with most studies in this systematic review observing unchanged rates of mitochondrial protein synthesis. Similarly, there were mixed results regarding the capacity for protein ingestion to enhance MPS responses compared with a placebo/non-protein control condition. For the most part, there was uniformity in the post-exercise measurement periods of MPS across all three models, with most studies incorporating analysis windows between 1 and 6 h post-exercise. As discussed previously, future studies that incorporate analysis windows of MPS that extend to 24–48 h post-exercise may provide greater resolution to identifying alterations in mitochondrial protein synthesis. Substantial variation in the exercise parameters utilised in the different study designs, particularly duration and relative intensity of exercise bouts, as well as in the type/quality and quantities of protein ingested, was noticeable across the three different models. Thus, the possibility that different exercise intensity and duration than those used in this systematic review can increase synthesis rates of mixed, myofibrillar, sarcoplasmic, or mitochondrial proteins cannot be excluded. Regarding protein provision, recent work identified a dose–response in myofibrillar protein synthesis in response to different amounts of protein ingestion following a session of continuous cycle exercise [61]. Specifically, 30 g and 45 g of milk protein ingestion elicited approximately 46% and approximately 52% greater MPS responses than no protein, respectively [61]. These findings fill a significant gap in the knowledge area as they indicate that 30 g is sufficient to maximise MPS rates after aerobic-based exercise. Although these findings are limited to young males and are in response to the specified bout of exercise, they show that greater amounts of protein may be required to maximally stimulate myofibrillar protein with aerobic-based exercise compared with resistance training. Notably, the same post-exercise protein dose–response increase was not observed with rates of mitochondrial protein synthesis. However, using intrinsic tracer labelling in this study, it was demonstrated that amino acids from ingested protein contribute to mitochondrial protein remodelling after endurance training [61]. Further focus on comparing how different types of protein (i.e., animal vs. plant-derived) and their ingestion distribution throughout the day can regulate MPS responses to aerobic-based exercise and HIIT remains an area of future study.

While there continues to be debate regarding the significance of MPS responses as they relate to their extrapolation to long-term changes in muscle hypertrophy [106], it still needs to be elucidated to what extent increases in MPS may benefit aspects of power or endurance performance with HIIT and aerobic-based exercise, respectively. As a potential avenue, this could entail correlating ‘high’ and ‘low’ responder changes in adaptation measures such as Wingate peak power or *V*O_2max_ with changes in MPS. While there are inherent logistical limitations of such work, the advent of deuterium oxide as a tracer for measuring MPS provides greater flexibility to measuring integrated rates of MPS over the course of several days and weeks that can factor in habitual activity and nutritional practices [107]. Nonetheless, the overwhelming capacity for both aerobic-based exercise and HIIT to significantly enhance post-exercise rates of MPS, as demonstrated by studies in this systematic review, should not be undervalued. While it is acknowledged that changes in MPS represent only one side of the overall net protein balance equation, increased MPS responses with these exercise modes are indicative of improved skeletal muscle quality, which, in the case of older adults or individuals with metabolic-related conditions (i.e., obesity, type 2 diabetes), can form the basis for improvements in muscle function and health.
